# Serum uromodulin is inversely associated with arterial hypertension and the vasoconstrictive prohormone CT-proET-1 in the population-based KORA F4 study

**DOI:** 10.1371/journal.pone.0237364

**Published:** 2020-08-07

**Authors:** Cornelia Then, Barbara Thorand, Holger L. Then, Christa Meisinger, Margit Heier, Annette Peters, Wolfgang Koenig, Wolfgang Rathmann, Martin Bidlingmaier, Andreas Lechner, Martin Reincke, Jürgen E. Scherberich, Jochen Seissler

**Affiliations:** 1 Medizinische Klinik und Poliklinik IV, Klinikum der Universität München, LMU München, Munich, Germany; 2 Clinical Cooperation Group Diabetes, Ludwig-Maximilians-Universität München and Helmholtz Zentrum München, Munich, Germany; 3 German Center for Diabetes Research (DZD), München-Neuherberg, Germany; 4 Institute of Epidemiology, Helmholtz Zentrum München–German Research Center for Environmental Health (GmbH), Neuherberg, Germany; 5 Freie Waldorfschule Augsburg, Augsburg, Germany; 6 Independent Research Group Clinical Epidemiology, Helmholtz Zentrum München–German Research Center for Environmental Health (GmbH), Neuherberg, Germany; 7 Chair of Epidemiology at UNIKAT Augsburg, Ludwig-Maximilians-Universität München, Munich, Germany; 8 KORA Study Centre, University Hospital Augsburg, Augsburg, Germany; 9 DZHK (German Centre for Cardiovascular Research), partner site Munich Heart Alliance, Munich, Germany; 10 Institute of Epidemiology and Medical Biometry, University of Ulm, Ulm, Germany; 11 Deutsches Herzzentrum München, Technische Universität München, Munich, Germany; 12 German Diabetes Center, Leibniz Institute at Heinrich Heine University Düsseldorf, Institute of Biometrics and Epidemiology, Düsseldorf, Germany; 13 Klinikum München-Harlaching, Teaching Hospital of the Ludwig-Maximilians-Universität, Munich, Germany; Anatomy, SWITZERLAND

## Abstract

**Objectives:**

Uromodulin has been associated with arterial hypertension in genome-wide association studies, but data from clinical and preclinical studies are inconsistent. We here analyzed the association of serum uromodulin (sUmod) with arterial hypertension and vasoactive hormones in a population-based study.

**Methods:**

In 1108 participants of the KORA F4 study aged 62–81 years, sUmod was measured and the association of sUmod with arterial hypertension was assessed using logistic regression models. The associations of sUmod with renin and aldosterone and with the vasoconstrictive prohormone C-terminal pro-endothelin-1 (CT-proET-1) were analyzed in 1079 participants and in 618 participants, respectively, using linear regression models.

**Results:**

After multivariable adjustment including sex, age, eGFR, BMI, fasting glucose, current smoking, previous stroke and myocardial infarction, sUmod was inversely associated with arterial hypertension (OR 0.78; 95% CI 0.68–0.91; p = 0.001). SUmod was not significantly associated with renin and aldosterone after adjustment for sex, age and eGFR. However, sUmod was inversely associated with CT-proET-1 (β -0.19 ± 0.04; p < 0.001) after adjustment for sex, age, eGFR, BMI, arterial hypertension, fasting glucose, current smoking, previous stroke and myocardial infarction. The association with CT-proET-1 was stronger in participants with hypertension (β -0.22 ± 0.04) than in normotensive participants (β -0.13 ± 0.06; p for interaction hypertension = 0.003 in the model adjusted for hypertension).

**Conclusions:**

SUmod was inversely associated with arterial hypertension and the vasoconstrictive prohormone CT-proET-1, suggesting direct or indirect effects of sUmod on blood pressure regulation.

## Introduction

Uromodulin is a glycosylphosphatidylinositol-anchored protein synthesized in tubular cells of the ascending limb of Henle´s loop. The vast majority of uromodulin is released into the urine by proteolytic cleavage [1–3]. Urinary uromodulin is crucial for renal integrity. Mutations of the uromodulin-coding gene (*UMOD*) may cause severe kidney disease, such as cystic kidney disease, recurring urinary tract infections, familial juvenile hyperuremic nephropathy and congenital nephrolithiasis [3–8]. A small proportion of uromodulin is actively secreted from the basolateral side of tubular cells into the interstitial space and circulation (serum uromodulin, sUmod) [9–11]. SUmod is considered to reflect intact tubular cells and thus indirectly nephron mass [11]. In line, sUmod emerged as a promising marker for kidney function [10, 12, 13]. The physiological role of uromodulin in the systemic circulation is still unknown. However, sUmod seems to play an important role beyond renal homeostasis, since it is inversely associated with all-cause and cardiovascular mortality [14–17] and with various cardiovascular risk factors, such as diabetes [18, 19], the metabolic syndrome and its single components including elevated blood pressure [20]. In contrast, genome-wide association studies [21–24] and animal models [25, 26] show an increased risk of arterial hypertension in uromodulin-increasing *UMOD* loci variants, and uromodulin has even been suggested to be a possible target for blood pressure control [26]. Decreased luminal translocation of the Na^+^/K^+^/2Cl^—^co-transporter (NKCC) in the thick ascending limb of Henle’s loop was proposed as a possible underlying mechanism [27]. However, upregulation of renin and compensatory responses in distal and proximal tubules were reported in an animal model as possible processes counteracting the impaired NKCC translocation [27]. Thus, preclinical evidence suggests an interaction of uromodulin with the vasoregulatory renin-angiotensin-aldosterone system [27].

A further potent vasoconstrictor, endothelin-1 (ET-1), which is stimulated by aldosterone in a negative feedback loop [28], also interferes with sodium and water uptake in the tubular system and collecting ducts. The 21-amino acid peptide ET-1 is secreted by vascular endothelium and by renal cells. The kidneys are both a source and a para- and autocrine target of ET-1. The renal medulla contains the highest concentration of ET-1 in the body and the tubular system may release more ET-1 than any other tissue [29]. ET-1 acts via different endothelin receptors (ETR) and mediates vasoconstrictive effects by targeting endothelial cells and vascular smooth muscle cells via ETR_A_ [30]. However, in the kidney, ET-1 predominantly binds to ETR_B_, mediating natriuresis [31].

The aim of the current study was to assess the association of sUmod with arterial hypertension in the population-based KORA F4 study. We further investigated the association of sUmod with renin and aldosterone as well as with the prohormone C-terminal pro-endothelin-1 (CT-proET-1), hypothesizing that these hormones are involved in an endocrine feedback regulation of blood pressure. ET-1 is instable and rapidly cleared from the circulation, preventing reliable measurements. Therefore, we used CT-proET-1, which is cleaved from the respective precursor protein during processing and secreted in equimolar concentrations, as stable surrogate parameter [32].

## Methods

### Study participants

The KORA (Cooperative Health Research in the Region of Augsburg) F4 study (2006–2008) is a follow-up examination of the population-based KORA S4 study (1999–2001). Recruitment and eligibility criteria for the KORA studies, study design, standardized sampling methods and data collection (medical history, medication, anthropometric measurements, blood pressure) have been described in detail previously [33–35]. The study was approved by the Ethics Committees of the Bavarian Medical Association (approval number 06068) in adherence to the declaration of Helsinki. All participants gave written informed consent before taking part. SUmod was measured in 1119 participants aged 62–81 years of the KORA F4 study with available serum samples (out of a total of 1161 participants in this age group). All variables necessary for the planned analyses were available in 1108 participants for the assessment of the association of sUmod with arterial hypertension and in 1097 participants for the assessment of the association of sUmod with renin/aldosterone. CT-proET-1 was measured in a subgroup (n = 618) participating in the first half of the KORA F4 study. Blood pressure was measured using a validated automatic device (OMRON HEM 705-CP). Three independent blood pressure measurements were taken in a sitting position on the right arm after a rest of at least 5 minutes with a 3-minute pause between measurements. Arterial hypertension was defined using the mean of the second and third blood pressure readings with a systolic blood pressure ≥ 140 mmHg and/or a diastolic blood pressure ≥ 90 mmHg, and/or intake of anti-hypertensive medication given that the participants were aware of having hypertension.

### Laboratory measurements

Blood was collected without stasis in sitting position after a rest of 10 minutes (sitting) and after an overnight fast of at least eight hours. Plasma and serum samples were assayed immediately or stored at -80°C. Measurements of serum creatinine was performed with the Jaffe method as described elsewhere [36]. Estimated glomerular filtration rate (eGFR) was calculated using the Chronic Kidney Disease Epidemiology Collaboration (CKD-EPI) equation (2009) based on serum creatinine [37]. SUmod was measured with a commercially available enzyme-linked immunosorbent assay kit (Euroimmun AG, Lübeck, Germany) with a lower detection limit of 2 ng/ml, an intra-assay coefficient of variation of 2.3% and inter-assay coefficients of variation of 4.4% and 9.5% for sUmod target values of 24.9 and 142.2 ng/ml, respectively. The measurement procedure was described by Steubl et al. [10]. Plasma concentrations of C-terminal pro-endothelin-1 (CT-proET-1) were measured by sandwich fluoroimmunoassay (BRAHMS, Hennigsdorf/Berlin, Germany) using the automated system B.R.A.H.M.S KRYPTOR as described previously [38]. The lower detection limit was 0.4 pmol/l. Plasma renin concentrations were measured using the Liaison active renin assay (Diasorin, Dietzenbach, Germany) using monoclonal antibodies to only detect active renin molecules without interference with pro-renin. Intra- and inter-assay coefficients of variation were below 5.6% and 12.2%, respectively, and the functional sensitivity was <2.0 μU/ml. Plasma aldosterone concentrations were determined with an inhouse immunoflurometric assay as described previously [39]. Inter- and intra-assay coefficients of variation were 15.2% and 7.3% in low, and 8.0% and 4.4% in high concentrations, respectively.

### Statistical analyses

The analyses were performed using the statistical environment R, version 3.6.3. Characteristics of the study participants were compared between participants without and with arterial hypertension using t-tests in case of normally distributed variables. Mann-Whitney U-tests were performed for variables with skewed distributions. Chi-square tests were used to compare binomial proportions. The associations of sUmod as independent variable with the outcomes of interest were assessed in logistic regression models in case of categorical dependent variables and in linear regression models in case of continuous dependent variables. Continuous variables were transformed to a Gaussian distribution by the probability integral transformation followed by an inverse transform sampling and were used as in calculations per one standard deviation. In logistic and linear regression analyses, the association of sUmod with the respective dependent variables was adjusted for covariates in 5 models (model 1: sex, age (which are both related to hypertension and have been shown to be associated with sUmod [18]); model 2: model 1 plus eGFR (which was decreased in hypertensive participants and is strongly related to sUmod [12]); model 3: model 2 plus BMI (which is associated with sUmod [20]), fasting glucose (associated with sUmod [18]), current smoking, previous stroke and myocardial infarction; model 4: model 3 plus arterial hypertension; model 5: model 4 plus use of beta-blockers, angiotensin-converting enzyme inhibitors and angiotensin receptor antagonists, since these medications might influence the renin-aldosterone-angiotensin system). Model 1 and 2 were applied for all analyses, model 3 for the assessment of arterial hypertension, model 4 for CT-proET-1 and model 5 for renin and aldosterone as dependent variables. The models are given in the tables for each observation. The level of statistical significance was set at 5% (two-sided), except for the hypertension interaction term, for which a p value < 0.10 was considered significant.

## Results

### Study population characteristics

Characteristics of the study population in total and stratified by arterial hypertension are shown in Table 1. Values for eGFR were 7.5% and for sUmod 21.9% lower in participants with arterial hypertension compared to normotensive participants. Renin and CT-proET-1 were significantly higher in participants with hypertension, whereas aldosterone was similar in both groups.

**Table 1:** Characteristics of study participants^a^.

	All participants	Participants without hypertension	Participants with hypertension	p ^b^
**n**	**1108**	**414**	**694**	
Female sex n (%)	546 (49)	230 (56)	316 (46)	< 0.001 ^###^
Age (years)	70.4 ± 5.5	69.0 ± 5.2	71.1 ± 5.5	< 0.001 ^#^
BMI (kg/m^2^)	28.7 ± 4.5	27.3 ± 4.0	29.6 ± 4.6	< 0.001 ^#^
Systolic blood pressure (mmHg)	128.5 ± 19.6	119.8 ± 13.8	133.7 ± 20.8	< 0.001 ^#^
Diastolic blood pressure (mmHg)	74.0 ± 10.0	71.8 ± 8.3	75.4 ± 10.7	< 0.001 ^#^
eGFR (ml/min/1.73 m^2^)	77.9 (67.3; 87.8)	81.4 (72.0; 89.7)	75.3 (64.2; 85.5)	< 0.001 ^##^
sUmod (ng/ml)	152.5 (110.1; 207.7)	176.9 (126.5; 228.4)	138.1 (99.2; 190.4)	< 0.001 ^##^
Previous stroke n (%)	46 (4)	5 (1)	41 (6)	0.21 ^###^
Previous myocardial infarction n (%)	65 (6)	16 (4)	49 (7)	< 0.001 ^###^
Current smoking n (%)	81 (7)	41 (10)	40 (6)	0.03 ^###^
**n**	**1097**	**409**	**688**	
Renin (ng/l)	11.0 (5.3; 22.4)	8.8 (5.1; 15.6)	12.8 (5.4; 30.9)	< 0.001 ^##^
Aldosterone (ng/l)	37.2 (24.0; 56.0)	38.0 (24.0; 56.0)	36.0 (25.2; 54.0)	0.81 ^##^
**n**	**618**	**220**	**398**	
CT-proET-1 (pmol/l)	51.5 (44.6; 61.2)	47.6 (42.8; 54.3)	53.8 (46.5; 64.3)	< 0.001 ^##^

^a^ Mean ± standard deviation, median (first quartile; third quartile), or number of participants (proportion in %)

^b^ The p value is related to the null hypothesis of no differences between participants with and without arterial hypertension

^#^ T-test

^##^Mann-Whitney U-test

^###^Chi-square test.

### Association of sUmod with arterial hypertension

SUmod was inversely associated with arterial hypertension in the crude analysis (OR 0.61; 95% CI 0.54–0.70; Table 2). Stepwise adjustment revealed an attenuation of the association of sUmod with arterial hypertension by correction for sex and age and eGFR. Adjustment for BMI further attenuated the OR to 0.76 (95% CI 0.66–0.88), but including fasting glucose, smoking, previous stroke and myocardial infarction into the model hardly affected the result, and the association remained significant in the fully adjusted model (OR 0.78; 95% CI 0.68–0.91; p = 0.001).

**Table 2:** Odds ratios (95% confidence interval) for arterial hypertension as dependent variable and sUmod as independent variable (per standard deviation).

Without adjustment	P value
0.61 (0.54; 0.70)	< 0.001
Model 1: Adjustment for sex and age	
0.66 (0.58; 0.76)	< 0.001
Model 2: Adjustment for sex, age and eGFR	
0.70 (0.61; 0.81)	< 0.001
Model 3: Adjustment for sex, age, eGFR, BMI, fasting glucose, smoking, previous stroke and myocardial infarction	
0.78 (0.68; 0.91)	0.001

Results of logistic regression models. n = 1108.

### Lacking association of sUmod with renin and aldosterone

In the crude analysis, sUmod was inversely related to renin in the total study group (β -0.14 ± 0.03; p < 0.001) and in participants with arterial hypertension (β -0.13 ± 0.04; p = 0.002). These associations were attenuated after adjustment for sex and age and lost significance after further adjustment for eGFR (Table 3). In a sensitivity analysis, we found an inverse association of renin with eGFR (β -0.18 ± 0.03; p < 0.001), which was stronger than the inverse association with sUmod, and a positive association of renin with male sex (β 0.41 ± 0.06; p < 0.001). There was no significant association of renin with age (β 0.05 ± 0.03; p = 0.12). The multivariable linear regression model including eGFR, sex and sUmod revealed a robust association of renin with eGFR (β -0.17 ± 0.03; p < 0.001) and with male sex (β 0.41 ± 0.06; p < 0.001), whereas the association with sUmod was attenuated to non-significance (β -0.04 ± 0.03; p = 0.18), indicating that the association of renin with sUmod was not independent of sex and eGFR. There was no significant association of sUmod with aldosterone in any model (Table 3).

**Table 3:** Association estimates between sUmod and renin and aldosterone.

	Renin	P value	Aldosterone	P value
Without adjustment				
Total study group	-0.14 ± 0.03	< 0.001	0.01 ± 0.10	0.857
No arterial hypertension	-0.08 ± 0.04	0.072	0.05 ± 0.05	0.332
Arterial hypertension	-0.13 ± 0.04	0.002	-0.02 ± 0.04	0.547
Model 1: Adjustment for sex and age				
Total study group	-0.10 ± 0.03	0.001	-0.02 ± 0.03	0.498
No arterial hypertension	-0.05 ± 0.04	0.261	0.04 ± 0.05	0.476
Arterial hypertension	-0.01 ± 0.04	0.024	-0.05 ± 0.04	0.176
Model 2: Adjustment for sex, age and eGFR				
Total study group	-0.05 ± 0.03	0.088	0.01 ± 0.03	0.699
No arterial hypertension	-0.04 ± 0.04	0.355	0.05 ± 0.05	0.375
Arterial hypertension	-0.04 ± 0.04	0.403	-0.01 ± 0.04	0.713
Model 5: Adjustment for sex, age, eGFR, BMI, fasting glucose, smoking, previous stroke and myocardial infarction, use of beta-blockers, angiotensin-converting enzyme inhibitors and angiotensin receptor antagonists (and arterial hypertension in the total study group)				
Total study group	0.02 ± 0.04	0.566	-0.02 ± 0.04	0.695
No arterial hypertension	-0.01 ± 0.04	0.895	0.08 ± 0.06	0.135
Arterial hypertension	0.01 ± 0.03	0.777	0.02 ± 0.04	0.456

β coefficient ± standard error from linear regression models are given per standard deviation sUmod. n = 1097 (total study group), 409 (without arterial hypertension), 688 (with arterial hypertension).

### Association of sUmod with CT-proET-1

SUmod was inversely associated with CT-proET-1 (Table 4). The association was more pronounced in participants with arterial hypertension (β -0.44 ± 0.05; p < 0.001) compared to participants without hypertension (β -0.21 ± 0.06; p < 0.001). Adjustment for sex and age only moderately attenuated these associations, whereas further adjustment for eGFR had a stronger impact on the observed β-coefficients in participants with hypertension. Nevertheless, the association of sUmod with CT-proET-1 remained significant in the fully adjusted model in participants with arterial hypertension (β -0.22 ± 0.04; p < 0.001) and in normotensive participants (β -0.13 ± 0.06; p = 0.023). Throughout the models, the association of sUmod with CT-proET-1 was stronger in hypertensive than in normotensive participants. The p value for interaction hypertension was 0.003 in a model adjusted for hypertension only, and 0.078 in the fully adjusted model.

**Table 4:** Association estimates between sUmod and CT-proET-1.

	CT-proET-1	P value
Without adjustment		
Total study group	-0.39 ± 0.04	< 0.001
No arterial hypertension	-0.20 ± 0.06	0.001
Arterial hypertension	-0.44 ± 0.05	< 0.001
P value for interaction hypertension		0.003
Model 1: Adjustment for sex and age		
Total study group	-0.34 ± 0.04	< 0.001
No arterial hypertension	-0.15 ± 0.06	0.014
Arterial hypertension	-0.40 ± 0.05	< 0.001
Model 2: Adjustment for sex, age and eGFR		
Total study group	-0.25 ± 0.03	< 0.001
No arterial hypertension	-0.16 ± 0.06	0.008
Arterial hypertension	-0.28 ± 0.04	< 0.001
Model 4: Adjustment for sex, age, eGFR, BMI, fasting glucose, smoking, previous stroke and myocardial infarction (and arterial hypertension in the total cohort)		
Total study group	-0.19 ± 0.04	< 0.001
No arterial hypertension	-0.13 ± 0.06	0.023
Arterial hypertension	-0.22 ± 0.04	< 0.001
P value for interaction hypertension		0.078

β coefficient ± standard error from linear regression models are given per standard deviation sUmod. n = 618 (total study group), 220 (without arterial hypertension), 398 (with arterial hypertension).

## Discussion

The current data demonstrates an inverse association of sUmod with arterial hypertension in a large population-based study. This finding strengthens the view that uromodulin mirrors a vaso- and nephroprotective mediator molecule. Our data are in line with previous clinical studies involving participants at high cardiovascular risk or with cirrhosis, which reported a lower prevalence of arterial hypertension in participants with higher serum or plasma uromodulin values [13, 14, 16, 40]. These findings are in contrast with the results of genome-wide association and preclinical studies. Single nucleotid polymorphisms in the *UMOD* promoter region increasing uromodulin levels are associated with hypertension [21–23, 41], and the *UMOD* rs13333226 minor G allele interrelates with a reduced urinary uromodulin excretion and a decreased risk of arterial hypertension [21]. In transgenic mice, *UMOD* overexpression correlates positively with blood pressure [26], and lower systolic blood pressure and resistance to salt-induced blood pressure changes were reported in *UMOD* -/- mice [25]. Liu et al. confirmed salt and water wasting in young *UMOD* -/- mice and identified an impaired luminal translocation of the NKCC-transporter in the thick ascending limb of Henle’s loop as probable underlying mechanism [27]. However, Liu et al. also showed an age-dependency of the *UMOD* knockout effect on arterial hypertension with oliguria and hypertension in aged *UMOD* -/- mice, possibly due to a marked upregulation of renin and compensatory responses in distal and proximal tubules [27]. In line, *UMOD* -/- mice in another study showed an upregulation of major distal ion transporters including Na^+^/K^+^-ATPase, NKCC2, chloride channel protein class K2, Na^+^/Cl^-^ channel and epithelial sodium channel [42]. In a further mouse model, genetic deletion of *UMOD* was reflected by a shift of Na^+^/Cl^−^ cotransporter activity from the early part of the distal convoluted tubule to the downstream segment [43]. Further, despite its relation to a decreased risk for arterial hypertension, the uromodulin-lowering *UMOD* rs13333226 G allele is associated with a lower fractional sodium excretion during liberal sodium intake [21], indicating an increased sodium reabsorption at the proximal tubular level in conditions of reduced uromodulin.

Aside from genetic variations, under physiological conditions, uromodulin possibly regulates blood pressure via interference with the tubular electrolyte and water transport [25, 26, 44]. Due to the glycosylphosphatidylinositol-anchor, urinary uromodulin can form a highly ordered organization constituting a physical water barrier on the luminal membrane of the epithelial cells of the thick ascending limb of Henle’s loop, thereby reducing water and salt reabsorption [44]. In line, a large study including more than 6500 participants found a positive relation of urinary uromodulin with sodium, chloride and potassium excretion [45]. Assuming uromodulin regulates blood pressure by decreasing the Na^+^/K^+^/Cl^—^reabsorption, low blood pressure should be associated with an activation of the renin-angiotensin-aldosterone system in conditions of low uromodulin. In our study, sUmod was inversely associated with renin, but this effect lost significance after multivariable adjustment. Further, there was no association with aldosterone levels. A possible explanation for the lack of an association of uromodulin with renin/aldosterone may be an interference of urinary uromodulin with the tubuloglomerular feedback via interaction with the NKCC2 at the macula densa under physiological conditions. Also, standard anti-hypertensive medication interfering with the renin-angiotensin-aldosterone system may have obscured the association in participants with hypertension.

SUmod was inversely associated with the vasoconstrictive prohormone CT-proET-1 in the current study. CT-proET-1 has been previously associated with renal function [46]. However, the association of sUmod with CT-proET-1 was largely independent of the eGFR. In the kidney, ET-1 also interferes with natriuresis. Binding of ET-1 to ETR_B_ inhibits NKCC activity in rats [31, 47]. Whether uromodulin and ET-1 directly interact in the kidneys to modulate electrolyte and water transport is not known. The effect of ETR_B_ depends on calcium influx, as shown in cultured renal inner medullary cells [48]. Uromodulin has recently been shown to influence calcium flux as it upregulates TRPV5 and TRPM6 and thus stimulates calcium and magnesium reabsorption [49, 50].

In our study, we measured uromodulin and CT-proET-1 in serum. In the urinary tract, uromodulin binds various components including leukocytes, fimbriated bacteria and calcium phosphate crystals [3]. It is not yet clarified whether sUmod has similar properties in the circulation and can bind, modulate, neutralize or eliminate serum components, including CT-proET-1, as well.

The relation of sUmod with CT-proET-1 was present in participants with or without arterial hypertension, but the effect was significantly stronger in hypertensive participants, indicating that a feed-back mechanism may exist in arterial hypertension to limit the action of the vasoconstrictive prohormone CT-proET-1. On the other hand, a decrease of sUmod secretion by paracrine actions of CT-proET-1 in the tubular system is also conceivable. Little is known about the regulation of uromodulin secretion into the circulation and mechanistic studies exploring the nature of the interaction of sUmod and vasoconstrictive hormones are lacking.

### Study limitations and strengths

Only participants aged ≥ 62 years were included. Thus, the association of sUmod with arterial hypertension and vasoactive hormones remains to be confirmed in a younger population. We only had measurements of uromodulin and CT-proET-1 in serum and cannot answer the question whether serum levels accurately reflect urine levels. In fact, uromodulin values in serum and in urine appear to correlate only moderately and their association with clinical characteristics may differ [51, 52]. Due to the observational nature of the study, we are not able to provide mechanistic links and causal relationships for our observations and reverse causation cannot be excluded. It is conceivable that hypertension-related microvascular injury may impair tubular function and thus decrease uromodulin secretion. Further, the impact of genetic polymorphisms on sUmod concentrations was not investigated in the current study. However, our analyses were done in a large, well characterized community-based study group. We measured sUmod with a sensitive and robust enzyme-linked immunosorbent assay. In contrast to uromodulin of urine origin, which forms various polymers with chancing epitopes and different antigenic sites [53], sUmod is a stable antigen without such pre-analytical disadvantages [9]. To our knowledge, the association of sUmod with the vasoconstrictive prohormone CT-proET-1 has not been analyzed before and the relation of sUmod with renin and aldosterone was not yet examined in humans.

## Conclusions

We demonstrate an inverse association of sUmod with arterial hypertension and with the vasoconstrictive prohormone CT-proET-1 in the population-based KORA F4 study. Thus, we provide indirect evidence for a systemic blood pressure regulative effect of sUmod. Further studies are required to clarify the mechanistic links between sUmod, arterial hypertension and ET-1.

## References

[1] Cavallone D, Malagolini N, Serafini-Cessi F. Mechanism of release of urinary Tamm-Horsfall glycoprotein from the kidney GPI-anchored counterpart. Biochem Biophys Res Commun. 2001;280: 110–114. 10.1006/bbrc.2000.4090 11162486

[2] Schaeffer C, Santambrogio S, Perucca S, Casari G, Rampoldi L. Analysis of Uromodulin Polymerization Provides New Insights into the Mechanisms Regulating ZP Domain-mediated Protein Assembly. Mol Biol Cell. 2008;20: 589–599. 10.1091/mbc.e08-08-0876 19005207 PMC2626557

[3] Devuyst O, Olinger E, Rampoldi L. Uromodulin: From physiology to rare and complex kidney disorders. Nature Reviews Nephrology. 2017 pp. 525–544. 10.1038/nrneph.2017.101 28781372

[4] Hart TC. Mutations of the UMOD gene are responsible for medullary cystic kidney disease 2 and familial juvenile hyperuricaemic nephropathy. J Med Genet. 2002;39: 882–892. 10.1136/jmg.39.12.882 12471200 PMC1757206

[5] Dahan K, Devuyst O, Smaers M, Vertommen D, Loute G, Poux JM, et al A Cluster of Mutations in the UMOD Gene Causes Familial Juvenile Hyperuricemic Nephropathy with Abnormal Expression of Uromodulin. J Am Soc Nephrol. 2003;14: 2883–2893. 10.1097/01.asn.0000092147.83480.b5 14569098

[6] Williams S, Reed AAC, Galvanovskis J, Antignac C, Goodship T, Karet FE, et al Uromodulin mutations causing familial juvenile hyperuricaemic nephropathy lead to protein maturation defects and retention in the endoplasmic reticulum. Hum Mol Genet. 2009;18: 2963–2974. 10.1093/hmg/ddp235 19465746 PMC2714724

[7] Bates JM, Raffi HM, Prasadan K, Mascarenhas R, Laszik Z, Maeda N, et al Tamm-Horsfall protein knockout mice are more prone to urinary tract infection. Kidney Int. 2004;65: 791–797. 10.1111/j.1523-1755.2004.00452.x 14871399

[8] Mo L, Huang HY, Zhu XH, Shapiro E, Hasty DL, Wu XR. Tamm-Horsfall protein is a critical renal defense factor protecting against calcium oxalate crystal formation. Kidney Int. 2004;66: 1159–1166. 10.1111/j.1523-1755.2004.00867.x 15327412

[9] Scherberich JE, Gruber R, Nockher WA, Christensen EI, Schmitt H, Herbst V, et al Serum uromodulin-a marker of kidney function and renal parenchymal integrity. Nephrology Dialysis Transplantation. 2018 pp. 284–295. 10.1093/ndt/gfw422 28206617 PMC5837243

[10] Steubl D, Block M, Herbst V, Nockher WA, Schlumberger W, Satanovskij R, et al Plasma uromodulin correlates with kidney function and identifies early stages in chronic kidney disease patients. Med (United States). 2016;95 10.1097/MD.0000000000003011 26962815 PMC4998896

[11] El-Achkar TM, Mccracken R, Liu Y, Heitmeier MR, Bourgeois S, Ryerse J, et al Tamm-Horsfall protein translocates to the basolateral domain of thick ascending limbs, interstitium, and circulation during recovery from acute kidney injury. Am J Physiol—Ren Physiol. 2013;304: 1066–1075. 10.1152/ajprenal.00543.2012 23389456 PMC3625838

[12] Then C, Then HL, Lechner A, Thorand B, Meisinger C, Heier M, et al Serum uromodulin and decline of kidney function in older participants of the population-based KORA F4/FF4 study. Clin Kidney J. 2020; 1–7. 10.1093/ckj/sfz154 33564420 PMC7857794

[13] Leiherer A, Muendlein A, Saely CH, Brandtner EM, Geiger K, Fraunberger P, et al The value of uromodulin as a new serum marker to predict decline in renal function. J Hypertens. 2018;36: 110–118. 10.1097/HJH.0000000000001527 28858977

[14] Delgado GE, Scherberich JE, Scharnagl H, Krämer BK, März W, Kleber ME. Serum Uromodulin and Mortality Risk in Patients Undergoing Coronary Angiography. J Am Soc Nephrol. 2017;28: 2201–2210. 10.1681/ASN.2016111162 28242751 PMC5491294

[15] Leiherer A, Muendlein A, Saely CH, Ebner J, Brandtner EM, Fraunberger P, et al Serum uromodulin is a predictive biomarker for cardiovascular events and overall mortality in coronary patients. Int J Cardiol. 2017;231: 6–12. 10.1016/j.ijcard.2016.12.183 28089453

[16] Steubl D, Buzkova P, Garimella PS, Ix JH, Devarajan P, Bennett MR, et al Association of serum uromodulin with mortality and cardiovascular disease in the elderly—the Cardiovascular Health Study. Nephrol Dial Transplant. 2019;gfz008 10.1093/ndt/gfz008 30903163 PMC7462724

[17] Then C, Then HL, Lechner A, Thorand B, Meisinger C, Heier M, et al Serum uromodulin and risk for cardiovascular morbidity and mortality in the community-based KORA F4 study. Atherosclerosis. 2020;297: 1–7. 10.1016/j.atherosclerosis.2020.01.030 32058862

[18] Then C, Then H, Meisinger C, Heier M, Peters A, Koenig W, et al Serum Uromodulin Is Associated With But Does Not Predict Type 2 Diabetes in Elderly KORA F4/FF4 Study Participants. J Clin Endocrinol Metab. 2019;104: 3795–3802. 10.1210/jc.2018-02557 30892596

[19] Leiherer A, Muendlein A, Saely CH, Kinz E, Brandtner EM, Fraunberger P, et al Serum uromodulin is associated with impaired glucose metabolism. Med (United States). 2017;96 10.1097/MD.0000000000005798 28151855 PMC5293418

[20] Then C, Then H, Lechner A, Huth C, Meisinger C, Heier M, et al Serum uromodulin is inversely associated with the metabolic syndrome in the KORA f4 study. Endocr Connect. 2019;8: 1363–1371. 10.1530/EC-19-0352 31505464 PMC6790901

[21] Padmanabhan S, Melander O, Johnson T, Di Blasio AM, Lee WK, Gentilini D, et al Genome-wide association study of blood pressure extremes identifies variant near UMOD associated with hypertension. PLoS Genet. 2010;6: e1001177 10.1371/journal.pgen.1001177 21082022 PMC2965757

[22] Van Eyk JE, Coresh J, Gudnason V, Hwang S-J, Fox CS, Larson MG, et al Uromodulin Levels Associate with a Common UMOD Variant and Risk for Incident CKD. J Am Soc Nephrol. 2009;21: 337–344. 10.1681/ASN.2009070725 19959715 PMC2834540

[23] Hixson JE, Köttgen A, Kao WHL, Hwang S-J, Shimmin LC, Schaeffer C, et al Association of Estimated Glomerular Filtration Rate and Urinary Uromodulin Concentrations with Rare Variants Identified by UMOD Gene Region Sequencing. PLoS One. 2012;7: e38311 10.1371/journal.pone.0038311 22693617 PMC3365030

[24] Köttgen A, Glazer NL, Dehghan A, Hwang SJ, Katz R, Li M, et al Multiple loci associated with indices of renal function and chronic kidney disease. Nat Genet. 2009;41: 712–7 10.1038/ng.377 19430482 PMC3039280

[25] Graham LA, Kumar S, Ferreri NR, Fraser NJ, Welsh P, Graham D, et al Validation of Uromodulin as a Candidate Gene for Human Essential Hypertension. Hypertension. 2013;63: 551–558. 10.1161/HYPERTENSIONAHA.113.01423 24324041

[26] Citterio L, Trudu M, Ikehata M, Dell’Antonio G, Demaretz S, Rampoldi L, et al Common noncoding UMOD gene variants induce salt-sensitive hypertension and kidney damage by increasing uromodulin expression. Nat Med. 2013;19: 1655–1660. 10.1038/nm.3384 24185693 PMC3856354

[27] Liu Y, Goldfarb DS, El-Achkar TM, Lieske JC, Wu X-R. Tamm-Horsfall protein/uromodulin deficiency elicits tubular compensatory responses leading to hypertension and hyperuricemia. Am J Physiol Physiol. 2018;314: F1062–F1076. 10.1152/ajprenal.00233.2017 29357410 PMC6032075

[28] Lynch IJ, Welch AK, Gumz ML, Kohan DE, Cain BD, Wingo CS. Effect of mineralocorticoid treatment in mice with collecting duct-specific knockout of endothelin-1. Am J Physiol—Ren Physiol. 2015;309: F1026–F1034. 10.1152/ajprenal.00220.2015 26400543 PMC4683304

[29] Dhaun N. The Endothelin System and Its Antagonism in Chronic Kidney Disease. J Am Soc Nephrol. 2006;17: 943–55. 10.1681/ASN.2005121256 16540557

[30] Kalani M. The importance of endothelin-I for microvascular dysfunction in diabetes. Vascular Health and Risk Management. 2008;4: pp. 1061–1068. 10.2147/vhrm.s3920 19183753 PMC2605330

[31] Ramseyer VD, Cabral PD, Garvin JL. Role of endothelin in thick ascending limb sodium chloride transport. Endothelin in Renal Physiology and Disease. 2011 pp. 76–83. 10.1159/000328686 21893990

[32] Papassotiriou J, Morgenthaler NG, Struck J, Alonso C, Bergmann A. Immunoluminometric assay for measurement of the C-terminal endothelin-I precursor fragment in human plasma. Clin Chem. 2006;52: 1144–1151. 10.1373/clinchem.2005.065581 16627560

[33] Holle R, Happich M, Löwel H, Wichmann HE. KORA—A research platform for population based health research. Gesundheitswesen. 2005;67: S19–25. 10.1055/s-2005-858235 16032513

[34] Rathmann W, Strassburger K, Heier M, Holle R, Thorand B, Giani G, et al Incidence of Type 2 diabetes in the elderly German population and the effect of clinical and lifestyle risk factors: KORA S4/F4 cohort study. Diabet Med. 2009;26: 1212–1219. 10.1111/j.1464-5491.2009.02863.x 20002472

[35] Meisinger C, Rückert IM, Rathmann W, Döring A, Thorand B, Huth C, et al Retinol-binding protein 4 is associated with prediabetes in adults from the general population: The cooperative health research in the Region of Augsburg (KORA) F4 study. Diabetes Care. 2011;34: 1648–1650. 10.2337/dc11-0118 21617096 PMC3120167

[36] Seissler J, Feghelm N, Then C, Meisinger C, Herder C, Koenig W, et al Vasoregulatory peptides pro-endothelin-1 and pro-adrenomedullin are associated with metabolic syndrome in the population-based KORA F4 study. Eur J Endocrinol. 2012;167: 847–853. 10.1530/EJE-12-0472 23002189

[37] Levey AS, Stevens LA, Schmid CH, Zhang Y, Castro AF, Feldman HI, et al A new equation to estimate glomerular filtration rate. Ann Intern Med. 2009;150: 604–612. 10.7326/0003-4819-150-9-200905050-00006 19414839 PMC2763564

[38] Morgenthaler NG, Struck J, Alonso C, Bergmann A. Assay for the measurement of copeptin, a stable peptide derived from the precursor of vasopressin. Clin Chem. 2006;52: 112–119. 10.1373/clinchem.2005.060038 16269513

[39] Manolopoulou J, Bielohuby M, Caton SJ, Gomez-Sanchez CE, Renner-Mueller I, Wolf E, et al A highly sensitive immunofluorometric assay for the measurement of aldosterone in small sample volumes: Validation in mouse serum. J Endocrinol. 2008;196: 215–224. 10.1677/JOE-07-0134 18252945

[40] Patidar KR, Garimella PS, Macedo E, Slaven JE, Ghabril MS, Weber RE, et al Admission plasma uromodulin and the risk of acute kidney injury in hospitalized patients with cirrhosis: a pilot study. Am J Physiol Gastrointest Liver Physiol. 2019;317: G447–G452. 10.1152/ajpgi.00158.2019 31411505 PMC6842992

[41] Köttgen A, Pattaro C, Böger CA, Fuchsberger C, Olden M, Glazer NL, et al New loci associated with kidney function and chronic kidney disease. Nat Genet. 2010;42: 376–84. 10.1038/ng.568 20383146 PMC2997674

[42] Bachmann S, Mutig K, Bates J, Welker P, Geist B, Gross V, et al Renal effects of Tamm-Horsfall protein (uromodulin) deficiency in mice. Am J Physiol Physiol. 2005;288: F559–67. 10.1152/ajprenal.00143.2004 15522986

[43] Tokonami N, Takata T, Beyeler J, Ehrbar I, Yoshifuji A, Christensen EI, et al Uromodulin is expressed in the distal convoluted tubule, where it is critical for regulation of the sodium chloride cotransporter NCC. Kidney Int. 2018;94: 701–715. 10.1016/j.kint.2018.04.021 30007527

[44] Padmanabhan S, Graham L, Ferreri NR, Graham D, McBride M, Dominiczak AF. Uromodulin, an emerging novel pathway for blood pressure regulation and hypertension. Hypertension. 2014 pp. 918–923. 10.1161/HYPERTENSIONAHA.114.03132 25110906

[45] Pruijm M, Ponte B, Ackermann D, Paccaud F, Guessous I, Ehret G, et al Associations of urinary uromodulin with clinical characteristics and markers of tubular function in the general population. Clin J Am Soc Nephrol. 2016;11: 70–80. 10.2215/CJN.04230415 26683888 PMC4702229

[46] Dhaun N, Yuzugulen J, Kimmitt RA, Wood EG, Chariyavilaskul P, MacIntyre IM, et al Plasma pro-endothelin-1 peptide concentrations rise in chronic kidney disease and following selective endothelin A receptor antagonism. J Am Heart Assoc. 2015;4: e001624 10.1161/JAHA.114.001624 25801761 PMC4392442

[47] Ramseyer VD, Ortiz PA, Carretero OA, Garvin JL. Angiotensin II-mediated hypertension impairs nitric oxide-induced NKCC2 inhibition in thick ascending limbs. Am J Physiol—Ren Physiol. 2016;310: F748–F754. 10.1152/ajprenal.00473.2015 26887831 PMC4835923

[48] Pandit MM, Inscho EW, Zhang S, Seki T, Rohatgi R, Gusella L, et al Flow regulation of endothelin-1 production in the inner medullary collecting duct. Am J Physiol—Ren Physiol. 2015;308: F541–F552. 10.1152/ajprenal.00456.2014 25587122 PMC4360032

[49] Wolf MTF, Wu XR, Huang CL. Uromodulin upregulates TRPV5 by impairing caveolin-mediated endocytosis. Kidney Int. 2013;84: 130–137. 10.1038/ki.2013.63 23466996 PMC3700562

[50] Nie M, Bal MS, Liu J, Yang Z, Rivera C, Wu XR, et al Uromodulin regulates renal magnesium homeostasis through the ion channel transient receptor potential melastatin 6 (TRPM6). J Biol Chem. 2018;293: 16488–16502. 10.1074/jbc.RA118.003950 30139743 PMC6200953

[51] Steubl D, Buzkova P, Ix JH, Devarajan P, Bennett MR, Chaves PHM, et al Association of serum and urinary uromodulin and their correlates in older adults—The Cardiovascular Health Study. Nephrology. 2020;25: 522–526. 10.1111/nep.13688 31846120 PMC7278530

[52] Risch L, Lhotta K, Meier D, Medina-Escobar P, Nydegger UE, Risch M. The serum uromodulin level is associated with kidney function. Clin Chem Lab Med. 2014;52: 1755–1761. 10.1515/cclm-2014-0505 24933630

[53] Youhanna S, Weber J, Beaujean V, Glaudemans B, Sobek J, Devuyst O. Determination of uromodulin in human urine: Influence of storage and processing. Nephrol Dial Transplant. 2014;29: 136–145. 10.1093/ndt/gft345 24097801

